# Contribution of Myelin Damage to White Matter Changes in Osmotic Demyelination Syndrome

**DOI:** 10.3390/diagnostics16050736

**Published:** 2026-03-01

**Authors:** Sung Ho Park, Young-Kwon Park, Jinwoo Choi, Minsu Ock, Dongseok Yang

**Affiliations:** 1 Department of Neurosurgery, Ulsan University Hospital, Ulsan 44033, Republic of Korea; michael@uuh.ulsan.kr; 2 Preventive Medicine Center, Ulsan University Hospital, Ulsan 44033, Republic of Korea; 0734714@uuh.ulsan.kr; 3 Departments of Physical Medicine & Rehabilitation, University of Ulsan College of Medicine, Ulsan University Hospital, Ulsan 44033, Republic of Korea; 0737190@uuh.ulsan.kr; 4 Departments of Preventive Medicine, University of Ulsan College of Medicine, Ulsan University Hospital, Ulsan 44033, Republic of Korea; ohohoms@naver.com

**Keywords:** diffusion tensor imaging, osmotic demyelination syndrome, axial diffusivity, radial diffusivity, myelin, white matter

## Abstract

**Background/Objectives:** Osmotic demyelination syndrome (ODS) causes marked myelin loss with relative axonal preservation. We used diffusion tensor imaging (DTI) to longitudinally assess white matter (WM) changes, hypothesizing that radial diffusivity (RD) would show dynamic recovery alongside clinical improvement. **Methods:** A 40-year-old woman with ODS and five age-matched female controls underwent DTI at 7 weeks and 6 months post-onset. Metrics were extracted from 27 WM tract categories using atlas-based regions of interest. Lesions were defined by directional dual thresholds (RD_d ≥ 2.0, axial diffusivity [AD] ≤ −2.0, or fractional anisotropy [FA] ≤ −2.0) and confirmed using the Crawford–Howell test with Benjamini–Hochberg FDR correction (q ≤ 0.05). Longitudinal percent change (Δ%) was compared using the Friedman test with Bonferroni-corrected Wilcoxon post hoc tests (α = 0.017). **Results:** Serum sodium increased from 126 to 138 mmol/L within 24 h, followed by a severe neurological deficit; near-complete recovery by 6 months. At 7 weeks, RD-defined lesions were detected in 10/27 tracts (37.0%)—1/6 brainstem-related and 9/21 non-brainstem—indicating widespread myelin-predominant injury. No AD- or FA-based lesions met criteria, although AD increase in the cingulate gyrus was significant. From 7 weeks to 6 months, the mean Δ% was −0.40 ± 9.38% (AD), −4.73 ± 9.73% (RD), and +7.94 ± 7.53% (FA). Changes differed across metrics (χ^2^(2) = 24.07, *p* = 5.92 × 10^−6^), with greater RD and FA changes than AD. **Conclusions:** Early RD-predominant abnormalities preceded RD reduction and FA increase during recovery, consistent with restoration of myelin-related microstructure. Larger studies are warranted.

## 1. Introduction

Osmotic demyelination syndrome (ODS) is a rare neurological disorder characterized by non-inflammatory demyelination that often spares axons [1,2]. It typically occurs following rapid correction of chronic hyponatremia, particularly in patients with alcoholism, liver disease, renal failure, or malnutrition [2,3,4]. ODS most commonly presents as central pontine myelinolysis (CPM), although extrapontine myelinolysis (EPM) or mixed forms may also occur. MRI studies have reported combined CPM and EPM in 25.4–44.6% of cases, with isolated EPM observed in 4.8–28.3% [1,4,5,6]. EPM lesions have been described in the cerebellum, lateral geniculate body, external and extreme capsules, hippocampus, putamen, and cortical and subcortical regions [6,7].

Although magnetic resonance imaging (MRI) is essential for diagnosing ODS, its sensitivity for detecting lesion severity and predicting clinical outcomes is limited [3,4,6,8,9]. Diffusion tensor imaging (DTI) is a quantitative MRI technique that enables evaluation of microstructural white matter (WM) integrity, including axonal and myelin status [10,11]. DTI-derived metrics, such as fractional anisotropy (FA), axial diffusivity (AD), and radial diffusivity (RD), reflect distinct pathological changes: decreases in AD are associated with axonal injury, whereas increases in RD indicate demyelination. Region of interest (ROI)-based DTI analyses have been widely used in conditions such as traumatic and anoxic brain injuries, Alzheimer’s disease, and various psychiatric disorders to characterize WM disruptions and correlate them with clinical outcomes [12,13,14,15,16,17]. Although ODS has been associated with widespread WM injury, only a few case studies have examined DTI parameters in this condition, and most assessed limited metrics, such as FA and mean diffusivity (MD) [18,19]. To the best of our knowledge, longitudinal tract-level DTI changes across global WM tracts in ODS have not been systematically evaluated using DTI-based WM parameters, including AD and RD.

In this study, we conducted a serial DTI analysis to investigate changes in FA, AD, and RD in a patient with ODS during recovery. We aimed to localize pontine and extrapontine tract lesions, differentiate between myelin and axonal damage, and evaluate the potential usefulness of DTI metrics for monitoring disease progression and neurological recovery.

## 2. Materials and Methods

### 2.1. Participants and Study Design

DTI metrics were analyzed at 7 weeks and 6 months post-onset in a 40-year-old woman with ODS and in five age-matched female control participants. The control group (mean age 41 ± 2.41 years) had no history of neuropsychiatric illness.

### 2.2. Clinical Data Collection

**Figure 1:**
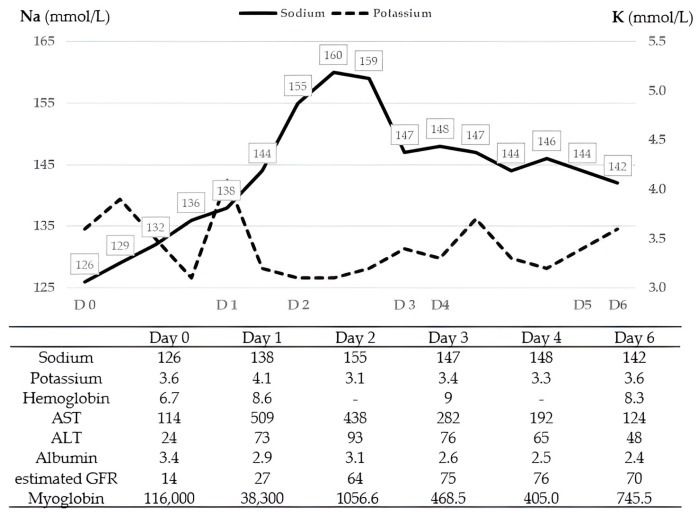
Serial laboratory changes during the initial hospitalization. The solid line represents serum Na (mmol/L; left *y*-axis), and the dashed line represents serum K (mmol/L; right *y*-axis) from day 0 to day 6. The table lists daily laboratory values, including hemoglobin (g/dL, reference: 12–16), AST (U/L, 0–40), ALT (U/L, 7–40), albumin (g/dL, 3.5–5.0), estimated GFR (mL/min/1.73 m^2^, >60), and urine myoglobin (ng/mL; <70). Abbreviations: ALT, alanine aminotransferase; AST, aspartate aminotransferase; D, day; GFR, glomerular filtration rate; K, potassium; Na, sodium.

The patient, who had alcoholic liver cirrhosis, presented with melena due to gastric varices and developed rhabdomyolysis-related acute kidney injury. On admission, her mental status and motor function were relatively intact; however, on the following day, she became comatose and completely paralyzed following rapid and fluctuating sodium correction (126 mmol/L on day 0, 138 mmol/L on day 1, 160 mmol/L on day 2, 147 mmol/L on day 3, and 142 mmol/L on day 6). These trends, along with other key laboratory parameters over the first week, are illustrated in Figure 1.

MRI was initially unremarkable (day 3) but subsequently showed pontine and bilateral internal capsule T2/fluid-attenuated inversion recovery hyperintensity at 2 and 5 weeks post-onset (Figure S1). When our department was consulted on day 18, the patient could partially follow one-step commands but exhibited severe dysarthria and a locked-in-like state without upper motor neuron signs. The progression of her clinical status over time is summarized in Table 1. DTIs were reviewed at 7 weeks (subacute phase) and 6 months (chronic recovery phase) to relate tract-level diffusion changes to clinical recovery.

### 2.3. MRI Acquisition and DTI Post-Processing

DTI scans were acquired on a 3.0 T scanner (Intera, Philips, The Netherlands) using a six-channel head coil and single-shot echo-planar imaging. For each of the 32 diffusion gradients, 80 contiguous slices parallel to the AC–PC line were obtained. All scans (patient and controls) were acquired on the same scanner using the same DTI protocol and parameters. The patient underwent DTI at two time points (7 weeks and 6 months post-onset), whereas each control underwent a single DTI scan (one time point per control; total control scans = 5).

Imaging parameters included: matrix size = 112 × 109, FOV = 224 × 224 mm^2^, TR/TE = 8973/80 ms, SENSE = 2, b = 1000 s/mm^2^, NEX = 2, and slice thickness = 2.0 mm. DTI data were processed using FSL software [20]. Head motion and eddy-current-induced distortions were corrected using FSL eddy, with corresponding rotation of the diffusion gradient table. The same preprocessing pipeline was applied to the patient and controls, with no group-specific preprocessing steps.

Susceptibility-induced distortion correction was not performed; therefore, residual EPI susceptibility distortion cannot be excluded, particularly near the skull base and within the pons/brainstem. For quality control, native-space diffusion images were visually inspected for gross warping or signal pile-up, and the mean FA map from the registered data was reviewed to confirm overall alignment (Figure S2). Nonetheless, subtle residual distortion may persist and could affect tract localization and ROI-based metric extraction.

ROIs were defined using the Johns Hopkins University (JHU) ICBM-DTI-81 atlas [15,21], selecting 48 major tracts. Using these methods, comprehensive coverage of pontine and extrapontine WM tracts was obtained. For tract-based statistical analyses, 48 individual WM tracts were extracted per scan and consolidated into 27 tract categories (21 averaged pairs and 6 unpaired). Accordingly, 54 tract observations were collected from the patient (27 tract categories × 2 time points [7 weeks and 6 months]) and 135 tract observations from the control group (27 tract categories × 5 participants). FA, AD, and RD values were analyzed; MD was not included because it is derived from AD and RD [11,16].

To examine pontine-related WM involvement, tracts were categorized as brainstem-related (including the pons) or non-brainstem-related based on the JHU atlas definitions. The brainstem-related group comprised six tracts: corticospinal tract, medial lemniscus, pontine crossing fibers, and the inferior, middle, and superior cerebellar peduncles; the remaining 21 tract categories were classified as non-brainstem-related.

### 2.4. Statistical Analyses

To assess early-stage, axon- and myelin-related injuries, tract-level abnormalities at 7 weeks were defined using a directional, dual-threshold single-case approach. For each tract and DTI metric, a directional standardized difference (effect size, d) was computed relative to control values (d = [patient value − control mean]/control standard deviation), and statistical deviation was assessed using the Crawford–Howell single-case test [22]. A tract–metric combination was classified as abnormal only if it met both criteria: (i) a prespecified directional effect size (RD_d ≥ 2.0, AD_d ≤ −2.0, or FA_d ≤ −2.0) using the Crawford–Howell single-case test, and (ii) Benjamini–Hochberg FDR-corrected significance for the Crawford–Howell test within the 7-week family of tests (q ≤ 0.05).

This dual-threshold approach combines a large effect-size cutoff (biological relevance) with FDR-corrected single-case significance (statistical reliability) to reduce false positives across multiple tract–metric tests. Lesion classification was directional and hypothesis-driven; therefore, non-lesion deviations—defined as tract–metric effects that were statistically significant (q ≤ 0.05) in the non-prespecified direction—were additionally reported descriptively.

For longitudinal analyses, percent change for each tract and metric was computed as Δ% = [(DTI at 6 months − DTI at 7 weeks)/DTI at 7 weeks] × 100%; therefore, recovery corresponds to FA increases and RD decreases. To compare longitudinal changes across metrics, Δ% values for AD, RD, and FA were compared using the Friedman test (repeated measures within tracts), followed by paired Wilcoxon signed-rank tests with Bonferroni correction (α = 0.05/3 = 0.017). Statistical analyses were performed in Jamovi (version 2.4.5).

## 3. Results

At the first DTI time point (7 weeks), the patient showed slight recovery after an early locked-in-like state, whereas substantial functional improvement was evident at the second time point (6 months; Table 1).

### 3.1. DTI Metrics and Lesion Distribution

**Figure 2:**
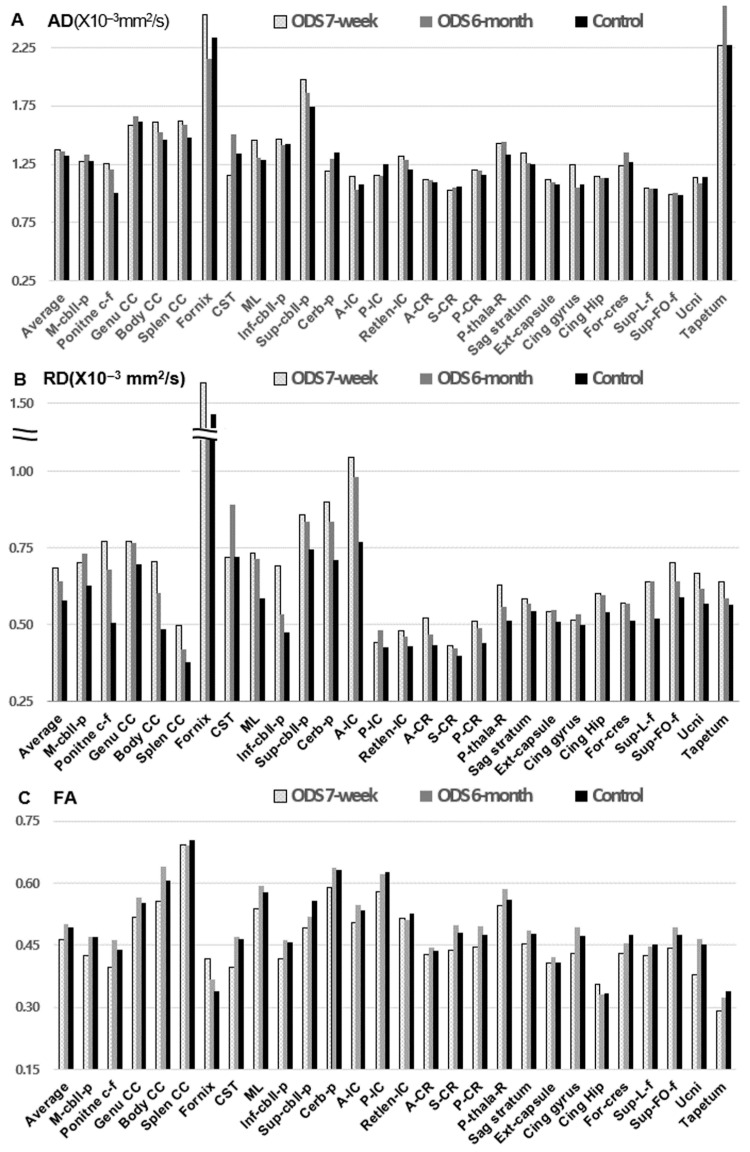
DTI metrics during recovery in a patient with ODS compared with a control population. DTI metrics for 27 tract categories at 7 weeks and 6 months after ODS onset, compared with those of age-matched controls. (**A**) AD (×10^−3^ mm^2^/s) values remained relatively stable between 7 weeks and 6 months and were comparable to control values across most tract categories. (**B**) RD (×10^−3^ mm^2^/s) was elevated at 7 weeks and showed a decreasing trend by 6 months, with values in several categories approaching those of the control group. (**C**) FA values showed an increase from 7 weeks to 6 months, moving closer to control levels across multiple tract categories. Abbreviations: A-CR, anterior corona radiata; A-IC, anterior limb of the internal capsule; AD, axial diffusivity; Body CC, body of the corpus callosum; Cerb-p, cerebral peduncle; Cing gyrus, cingulum (cingulate gyrus); Cing Hip, cingulum (hippocampal part); CST, corticospinal tract; DTI, diffusion tensor imaging; Ext-capsule, external capsule; FA, fractional anisotropy; For-cres, fornix (cres)/stria terminalis; Genu CC, genu of the corpus callosum; Inf-cbll-p, inferior cerebellar peduncle; M-cbll-p, middle cerebellar peduncle; ML, medial lemniscus; ODS, osmotic demyelination syndrome; P-CR, posterior corona radiata; P-IC, posterior limb of the internal capsule; P-thala-R, posterior thalamic radiation; Pontine c-f, pontine crossing fibers; RD, radial diffusivity; Retlen-IC, retrolenticular part of the internal capsule; S-CR, superior corona radiata; Sag stratum, sagittal stratum; Splen CC, splenium of the corpus callosum; Sup-cbll-p, superior cerebellar peduncle; Sup-FO-f, superior fronto-occipital fasciculus; Sup-L-f, superior longitudinal fasciculus; Ucni, uncinate fasciculus.

The patient’s DTI values, averaged across 27 WM tract categories (21 bilateral paired tracts averaged across hemispheres and 6 unpaired tracts), were as follows: AD (×10^−3^ mm^2^/s) remained stable (1.37 ± 0.37 at 7 weeks and 1.36 ± 0.38 at 6 months post onset) compared with controls (1.32 ± 0.34). RD (×10^−3^ mm^2^/s) decreased from 0.68 ± 0.23 at 7 weeks to 0.64 ± 0.18 at 6 months, approaching the control value (0.58 ± 0.20). FA increased from 0.46 ± 0.08 at 7 weeks to 0.50 ± 0.09 at 6 months, compared with controls (0.49 ± 0.09; Figure 2).

Tract-level lesions at 7 weeks were defined using the FDR-corrected directional dual-threshold approach: increased RD (RD_d > 2.0 and q < 0.05) was interpreted as a myelin-predominant abnormality, whereas decreased AD or FA (AD_d < −2.0 or FA_d < −2.0, both with q ≤ 0.05) was interpreted as an axonal or tract-integrity abnormality. Using this definition, primary lesions were detected only in RD: 10 of 27 tracts (37.0%) met criteria for RD-defined abnormalities (Table 2). No tracts met criteria for AD- or FA-based lesions, but the cingulate gyrus showed a markedly increased AD (AD_d = 6.27, q = 0.0421), which is reported as a non-lesion deviation rather than being classified as a hypothesis-consistent lesion (Table S1).

By anatomical distribution, RD-defined lesions involved 1 of 6 brainstem-related tracts and 9 of 21 non-brainstem tract categories, indicating widespread RD abnormalities extending beyond the pons.

### 3.2. Longitudinal Changes in DTI Metrics

Paired raw differences in DTI metrics (Δ = 6 months − 7 weeks) and percent changes are summarized as follows. Across the 27 tract categories, the mean raw differences were ΔAD = −0.009 ± 0.151 (×10^−3^ mm^2^/s), ΔRD = −0.041 ± 0.097 (×10^−3^ mm^2^/s), and ΔFA = 0.036 ± 0.032, with corresponding medians [interquartile ranges, IQR] of −0.020 [0.085], −0.020 [0.065], and 0.040 [0.025], respectively. Across the 27 tract categories, the mean percent changes from 7 weeks to 6 months post-onset were ΔAD = −0.40 ± 9.38%, ΔRD = −4.73 ± 9.73%, and ΔFA = 7.94 ± 7.53% (median [IQR]: −1.74 [6.31], −4.09 [9.00], and 8.89 [7.38], respectively).

Because ΔAD, ΔRD, and ΔFA are repeated measures within the same tract units, differences among metrics were assessed using the Friedman test, which showed a significant overall effect (χ^2^(2) = 24.07, *p* = 5.92 × 10^−6^). Post hoc paired Wilcoxon signed-rank tests with Bonferroni correction (α = 0.017) showed significant differences between ΔRD and ΔAD (W = 88, *p* = 0.014), between ΔFA and ΔAD (W = 45, *p* = 2.36 × 10^−4^), and between ΔFA and ΔRD (W = 13, *p* = 1.31 × 10^−6^; Figure S3). Collectively, these results indicate that longitudinal evolution was driven primarily by FA recovery and RD normalization, whereas AD remained comparatively stable.

## 4. Discussion

This study identified widespread myelin injury with relative axonal preservation in a patient with ODS, based on atlas-based quantitative and longitudinal DTI analyses. Severe early neurological impairment does not preclude near-complete recovery [23,24]. Two large cohort studies have reported that approximately 60% of patients regain functional independence (modified Rankin Scale score 0–2) within 3–6 months after ODS [3,4]. In addition, conventional MRI lesion characteristics (e.g., lesion volume and pontine/extrapontine involvement) and traditional risk factors, such as alcoholism and liver disease, are not consistently associated with long-term functional outcomes [3,4,8,9]. These observations highlight the need for more sensitive imaging measures, including serial DTI metrics, to better reflect ODS pathologic findings and their evolution during recovery.

At 7 weeks post-onset, when the patient had severe neurological deficits, 37.0% of WM tract categories (10/27) met lesion criteria based on increased RD, indicating widespread myelin-predominant injury. This pattern involved 16.7% of brainstem-related tract categories (1/6) and 42.9% of non-brainstem tract categories (9/21), which is compatible with established radiological findings of ODS affecting both pontine and extrapontine regions [1,4,5,6,7]. By 6 months post-onset, RD showed a significantly larger longitudinal decrease than AD, whereas AD exhibited comparatively smaller changes across tract categories. This metric-specific pattern is consistent with alterations in myelin-related microstructural properties. These findings are consistent with prior postmortem studies identifying myelin destruction as the hallmark of ODS, with axonal structures largely preserved. Wright et al. reported demyelination in both pontine and extrapontine regions in all 11 autopsy cases [25], and Newell and Kleinschmidt-DeMasters described myelin loss with axonal preservation in 14 of 15 autopsy cases [26]. Although these studies provided foundational knowledge of ODS pathological features, in vivo imaging evidence confirming this selective damage has remained limited. Notably, we observed an isolated AD increase in the cingulate gyrus, which is not the expected pattern for axonal injury (typically associated with AD decreases). Given the single-case design, we classified this finding as a non-lesion deviation because measurements in curved tracts adjacent to cerebrospinal fluid are vulnerable to geometric and partial-volume effects [11].

Two of three prior DTI studies analyzed only a few tracts in patients with chronic ODS, using FA and MD [18,19]. However, these approaches have inherent limitations. First, MD and apparent diffusion coefficient metrics are not specific for distinguishing myelin from axonal damage. Second, although ODS lesions involve both pontine and extrapontine regions, only a limited number of WM tracts were analyzed, such as the corticospinal, anterior thalamic, and pontocerebellar tracts. Finally, operator bias may have occurred because DTI metrics were derived from operator-defined ROIs. A recent study of acute-stage CPM assessed all DTI metrics in two patients with initial locked-in syndrome who recovered over the following 12 months [23]. DTI showed lower FA and higher RD, AD, and MD than in controls, but imaging was performed at non-matched time points corresponding to markedly different clinical stages (severe paralysis at 20 days vs. near-intact motor function at 5 months), limiting interpretability. This pattern is difficult to reconcile with pathological evidence of predominant demyelination with relative axonal preservation, underscoring limitations of prior DTI approaches. Our atlas-based serial analysis across global WM tracts provides a broader and more objective assessment of injury and recovery.

Compared with other acquired brain injuries, this single-case ODS trajectory is more consistent with a myelin-predominant pattern. Traumatic brain injury and stroke over similar subacute-to-chronic intervals with favorable outcomes typically show concurrent increases in AD and variable changes in FA and RD, reflecting combined axonal and myelin alterations [27,28,29]. In contrast, our findings were characterized by RD elevation with relative AD stability and partial RD normalization during recovery, a pattern consistent with improvement in myelin-related microstructural integrity rather than primary axonal disruption, although it does not directly demonstrate remyelination [27,29,30]. Differences in outcome definitions, tract selection, and methodological challenges may also contribute to variability across studies [27,29,30]. Collectively, this comparison underscores that ODS may exhibit a distinctive DTI evolution in which RD changes are more prominent than AD changes, aligning with the known pathological features of the disorder.

This study has several limitations. First, it is a single-patient, hypothesis-generating longitudinal analysis with a small normative reference sample; therefore, lesion counts and tract-level inferences should be interpreted cautiously and may not generalize to the broader ODS population. Second, although we used a DTI-based WM atlas, DTI metrics (including RD and AD) are indirect markers of microstructure, and longitudinal changes may reflect not only myelin- or axon-related processes but also edema/inflammation, gliosis, fiber reorganization, crossing fibers, and partial-volume effects. Third, susceptibility-induced distortion correction was not performed; thus, residual geometric distortion cannot be excluded and may have influenced tract localization and ROI-based metric extraction, particularly in regions prone to EPI susceptibility effects (e.g., near the skull base and brainstem/pons). Accordingly, findings involving pontine and brainstem tracts should be interpreted cautiously and were not used as the sole basis for biological inference.

## 5. Conclusions

This preliminary single-case longitudinal DTI analysis demonstrated RD-predominant tract abnormalities in ODS, followed by subsequent RD decreases and FA increases during clinical recovery, while AD remained relatively stable. This pattern is compatible with myelin-related microstructural improvement but is not specific to remyelination. These hypothesis-generating findings suggest that atlas-based tract-level DTI may help quantify ODS-related changes and warrant validation in larger cohorts.

## Figures and Tables

**Table 1:** Clinical manifestation over time.

	Time After Onset		
	5 Weeks	7 Weeks	6 Months
Cognition			
MMSE (max 30)/IQ	0/-	21/-	29/66
Manual motor grade			
Upper extremity (R/L)	2/3	3/3	5/5
Lower extremity (R/L)	2/3	3/3	5/5
Hand function			
MBC (1–6)	3/3	5/4	6/6
Grip power (kg)	0/0	7/0	14.3/2.7
Pegboard test (R/L)	0/0	0/0	9.3/9.0
Mobility			
SUB (0–4)	0	4	4
FAC (0–5)	0	1	4
MBI (0–100)	0	48	91

Abbreviations: FAC, Functional Ambulatory Category; IQ, Wechsler Adult Intelligence Scale; L, left; MBC, Modified Brunnstrom Classification; MBI, Modified Barthel Index; MMSE, Mini-Mental State Examination; R, right; SUB, Sitting Unsupported (Berg Balance Scale).

**Table 2:** Tract-level lesion classification using the directional, dual-threshold criterion at 7 weeks post-onset.

Region	No. Tracts	RD Lesions (RD↑)	AD Lesions (AD↓)	FA Lesions (FA↓)
Brainstem	6	1/6 (16.7%)	0/6 (0%)	0/6 (0%)
Non-brainstem	21	9/21 (42.9%)	0/21 (0%)	0/21 (0%)
Total	27	10/27 (37.0%)	0/27 (0%)	0/27 (0%)

Proportions represent tracts meeting the dual-threshold criterion for lesions (|d| ≥ 2.0 and Benjamini–Hochberg FDR-corrected Crawford–Howell q ≤ 0.05) for the indicated direction of change. Note: AD↓ = significant decrease, FA↓ = significant decrease, RD↑ = significant increase, all relative to controls. Non-lesion deviation, defined as significant effects (FDR-corrected q ≤ 0.05) occurring in the non-prespecified direction, is not counted as directional lesions (Cingulate gyrus, AD_d = 6.27, q = 0.0421). Abbreviations: AD, axial diffusivity; RD, radial diffusivity; FA, fractional anisotropy; d, standardized difference; FDR, false discovery rate; q, FDR-adjusted *p*-value.

## Data Availability

Data for these analyses are available upon reasonable request to the corresponding author.

## Supplementary Materials

The following supporting information can be downloaded at: https://www.mdpi.com/article/10.3390/diagnostics16050736/s1. Figure S1: Serial MRI findings at 2 weeks and 5 weeks post-onset; Figure S2: Mean FA map from the registered diffusion data (quality control); Figure S3: Magnitude of longitudinal changes across diffusion metrics; Table S1: Tract-level lesion classification using the directional, dual-threshold criterion at 7 weeks post-onset.

## Abbreviations

The following abbreviations are used in this manuscript:

AD	axial diffusivity
CPM	central pontine myelinolysis
DTI	diffusion tensor imaging
EPI	echo-planar imaging
EPM	extrapontine myelinolysis
FA	fractional anisotropy
FDR	false discovery rate
IQR	interquartile range
JHU	Johns Hopkins University
MD	mean diffusivity
MRI	magnetic resonance imaging
ODS	osmotic demyelination syndrome
RD	radial diffusivity
ROI	region of interest
WM	white matter
