# Supplementary material for: Contribution of Myelin Damage to White Matter Changes in Osmotic Demyelination Syndrome
Source: Diagnostics (Basel). 2026 Mar 1;16(5):736. doi: 10.3390/diagnostics16050736 (PMC12985121; doi:10.3390/diagnostics16050736)
Supplement: Supplementary file 1 [file diagnostics-16-00736-s001.zip › R_minor_Supplementary figure-table.pdf]

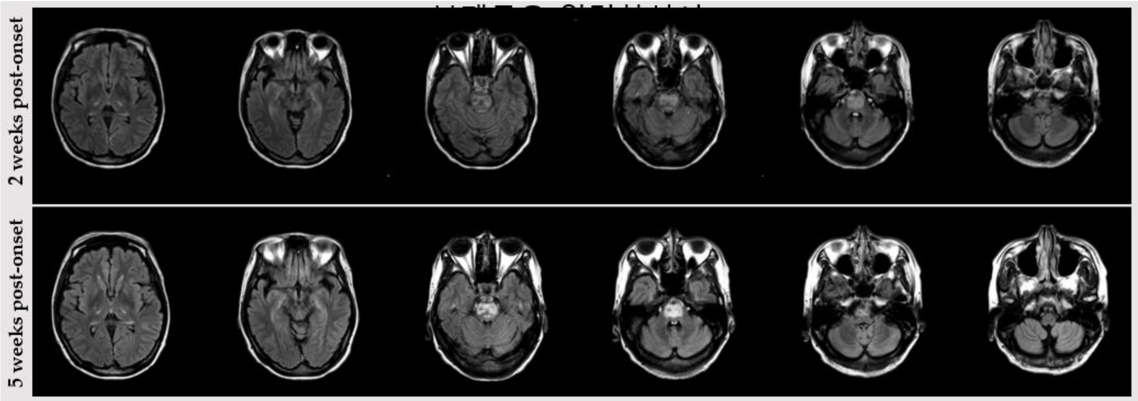

Figure S1. Serial MRI findings at 2 weeks and 5 weeks post-onset. Initial MRI at day 3 was unremarkable (not shown). The T2-weighted and FLAIR images on day 3 post-onset are unremarkable. At 2 weeks and 5 weeks post-onset, the MRI shows a high signal in the entire pons and symmetrical high signals in the posterior limb of the internal capsules and thalamus in both hemispheres, suggesting central pontine and extrapontine myelinolysis.
Abbreviation: MRI, magnetic resonance imaging; FLAIR, fluid-attenuated inversion recovery.

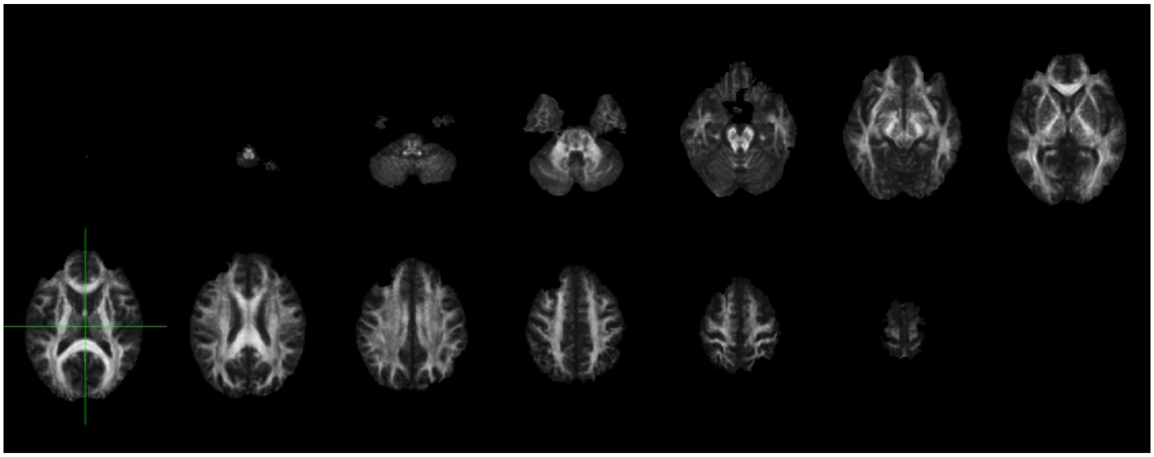

Figure S2. Mean fractional anisotropy (FA) map from the registered diffusion data (quality control). Visual inspection suggests overall anatomical alignment at the pons/brainstem level without obvious gross geometric warping or systematic displacement; however, subtle residual susceptibility distortion cannot be excluded.
Abbreviations: FA, fractional anisotropy.

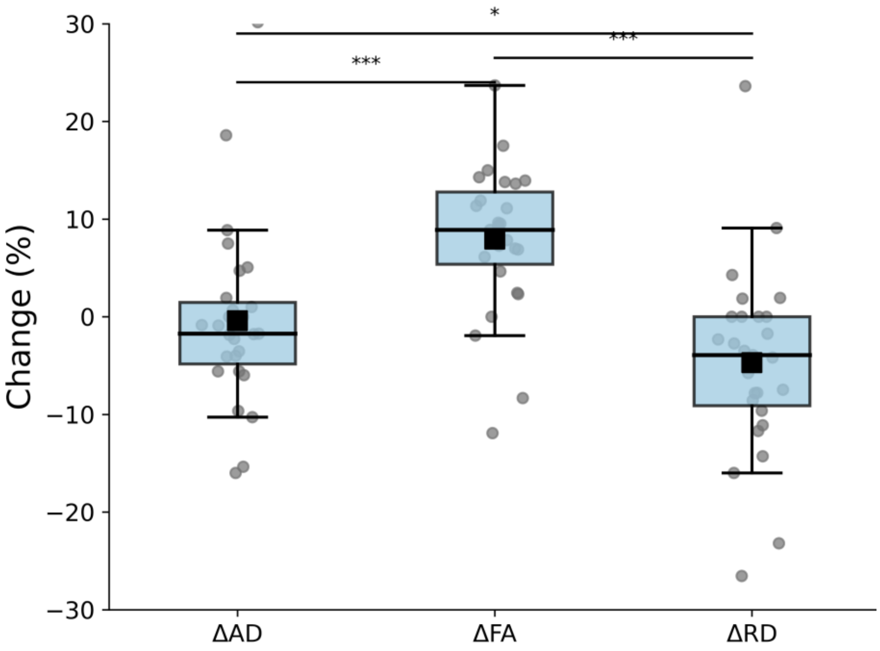

Figure S3. Magnitude of longitudinal changes across diffusion metrics. Percent change for each tract was computed as **Δ% =** (6 months − 7 weeks) / (7 weeks) × 100% for AD, FA, and RD (n = 27 tracts). Boxplots show the median and IQR, with whiskers extending to 1.5× IQR; each dot represents one tract, and squares indicate the mean. Because ΔAD, ΔFA, and ΔRD are repeated measures within the same tract units, differences among metrics were assessed using the Friedman test ($\chi^2(2) = 24.69$, $p = 4.35 \times 10^{-6}$), followed by paired Wilcoxon signed-rank tests with Bonferroni correction (FA vs AD: $p = 2.36 \times 10^{-4}$; FA vs RD: $p = 2.04 \times 10^{-6}$; RD vs AD: $p = 0.0176$).
Abbreviations: AD, axial diffusivity; FA, fractional anisotropy; IQR, interquartile range; RD, radial diffusivity.

Table S1. Tract-level lesion classification using the directional, dual-threshold criterion at 7 weeks post-onset.

Tract category	AD (d, p, q)	RD (d, p, q)	FA (d, p, q)	Directional lesion (FDR)	Non-lesion deviation (q ≤ 0.05)
**M-cbll-p**	**-0.17, 0.8855, 0.9563**	**1.81, 0.1738, 0.3911**	**-1.35, 0.2862, 0.5201**	**No lesion**	**—**
**Pontine c-f**	**4.03, 0.0212, 0.1134**	**9.01, 0.0012, 0.0304**	**-0.52, 0.6584, 0.8338**	**Myelin (RD↑)**	**—**
Genu CC	-0.87, 0.4703, 0.6876	3.74, 0.0270, 0.1249	-0.48, 0.6866, 0.8338	No lesion	—
Body CC	3.25, 0.0414, 0.1597	6.97, 0.0031, 0.0418	-0.62, 0.6018, 0.7609	Myelin (RD↑)	—
Splen CC	1.46, 0.2521, 0.4973	11.11, 0.0005, 0.0284	0.03, 0.9761, 0.9848	Myelin (RD↑)	—
Fornix	1.54, 0.2316, 0.4816	0.94, 0.4397, 0.6757	1.84, 0.1675, 0.3911	No lesion	—
**CST**	**-1.81, 0.1733, 0.3911**	**-0.02, 0.9848, 0.9848**	**-0.97, 0.4269, 0.6403**	**No lesion**	**—**
**ML**	**2.02, 0.1387, 0.3605**	**3.13, 0.0462, 0.1733**	**-0.25, 0.8292, 0.9221**	**No lesion**	**—**
**Inf-cbll-p**	**0.74, 0.5341, 0.7609**	**4.03, 0.0212, 0.1134**	**-0.98, 0.4227, 0.6403**	**No lesion**	**—**
**Sup-cbll-p**	**4.92, 0.0109, 0.0790**	**2.88, 0.0582, 0.2018**	**-1.13, 0.3601, 0.6209**	**No lesion**	**—**
Cerb-p	-2.85, 0.0598, 0.2018	6.22, 0.0048, 0.0421	-1.64, 0.2094, 0.4240	Myelin (RD↑)	—
A-IC	1.84, 0.1687, 0.3911	7.82, 0.0020, 0.0324	-0.36, 0.7620, 0.8817	Myelin (RD↑)	—
P-IC	-1.86, 0.1651, 0.3911	5.65, 0.0067, 0.0493	-1.29, 0.3036, 0.5201	Myelin (RD↑)	—
Retlen-IC	1.94, 0.1512, 0.3765	4.07, 0.0206, 0.1134	-0.03, 0.9814, 0.9848	No lesion	—
A-CR	0.80, 0.5065, 0.6954	6.05, 0.0052, 0.0421	-0.21, 0.8540, 0.9476	Myelin (RD↑)	—
S-CR	-1.05, 0.3906, 0.6403	4.81, 0.0117, 0.0790	-1.75, 0.1862, 0.3911	No lesion	—
P-CR	1.73, 0.1890, 0.3911	6.44, 0.0042, 0.0421	-0.99, 0.4178, 0.6403	Myelin (RD↑)	—
P-thala-R	4.13, 0.0196, 0.1134	8.44, 0.0015, 0.0304	-0.61, 0.6058, 0.7609	Myelin (RD↑)	—
Sag stratum	1.55, 0.2290, 0.4816	2.56, 0.0795, 0.2509	-0.51, 0.6644, 0.8338	No lesion	—

Ext-capsule	0.99, 0.4155, 0.6757	2.29, 0.1048, 0.3036	0.41, 0.7293, 0.8817	No lesion	—
Cing gyrus	6.27, 0.0046, 0.0421	0.85, 0.4804, 0.6876	-0.28, 0.8140, 0.9221	No lesion	AD↑
Cing Hip	0.17, 0.8854, 0.9563	2.48, 0.0867, 0.2601	0.99, 0.4171, 0.6403	No lesion	—
For-cres	-0.60, 0.6106, 0.8338	2.12, 0.1249, 0.3354	-1.15, 0.3536, 0.6209	No lesion	—
Sup-L-f	0.13, 0.9129, 0.9734	10.30, 0.0007, 0.0284	-0.71, 0.5537, 0.7609	Myelin (RD↑)	—
Sup-FO-f	-0.09, 0.9387, 0.9734	4.34, 0.0167, 0.1085	-0.72, 0.5451, 0.7609	No lesion	—
Ucni	-0.08, 0.9460, 0.9734	4.41, 0.0158, 0.1085	-1.59, 0.2200, 0.4240	No lesion	—
Tapetum	0.82, 0.4950, 0.6876	2.69, 0.0698, 0.2433	-1.77, 0.1818, 0.3911	No lesion	—

Notes: Directional lesions were defined by a dual-threshold criterion requiring (i) a prespecified directional effect size (RD_d ≥ 2.0, AD_d ≤ −2.0, or FA_d ≤ −2.0) and (ii) Benjamini–Hochberg FDR–corrected Crawford–Howell significance within the 7-week family of tract–metric tests (q ≤ 0.05). Non-lesion deviation, defined as significant effects (FDR-corrected q ≤ 0.05) occurring in the non-prespecified direction, is not counted as directional lesions (Cingulate gyrus, AD_d = 6.27, q = 0.0421). Boldface indicates brainstem-related tracts.

Abbreviations: AD, axial diffusivity; d, standardized difference; FA, fractional anisotropy; FDR, false discovery rate; p, Crawford–Howell p value; q, FDR-adjusted p value; RD, radial diffusivity. The tract abbreviations are defined in the legend of Figure 2.
