# Supplementary figures and images for: Contribution of Myelin Damage to White Matter Changes in Osmotic Demyelination Syndrome
Source: Diagnostics (Basel). 2026 Mar 1;16(5):736. doi: 10.3390/diagnostics16050736 (PMC12985121; doi:10.3390/diagnostics16050736)

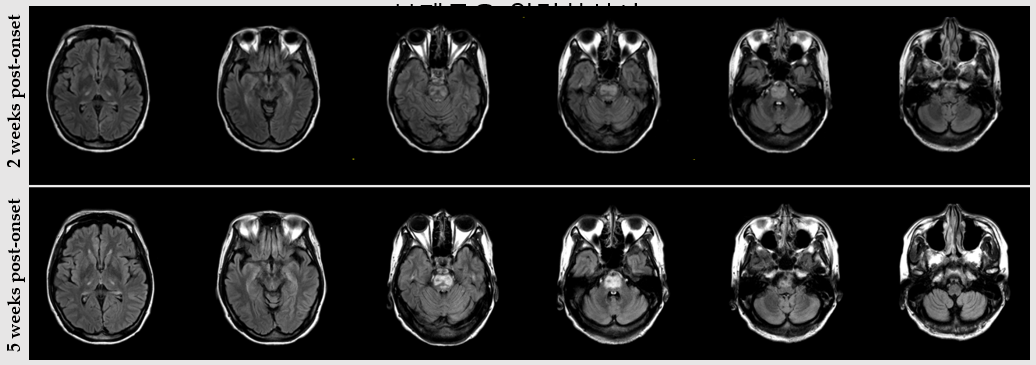

Supplement: Supplementary file 1 [file diagnostics-16-00736-s001.zip › Figure S1.png]

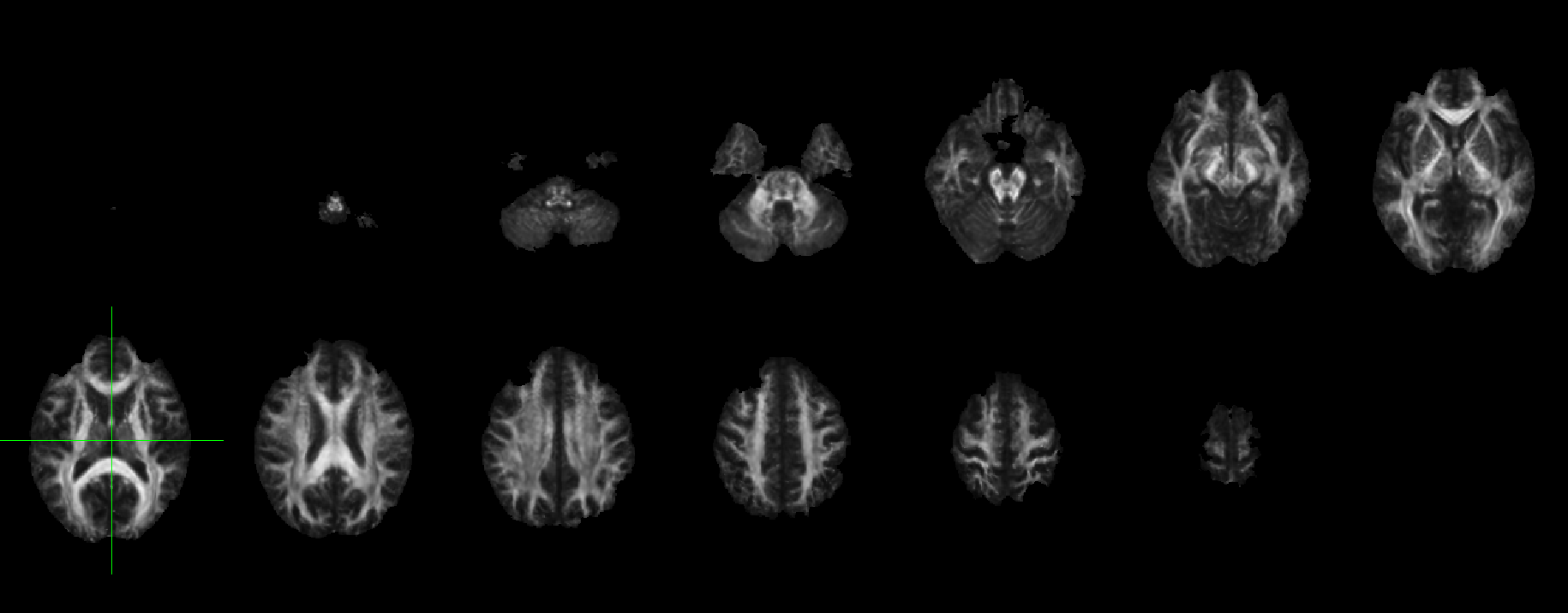

Supplement: Supplementary file 1 [file diagnostics-16-00736-s001.zip › Figure S2.png]

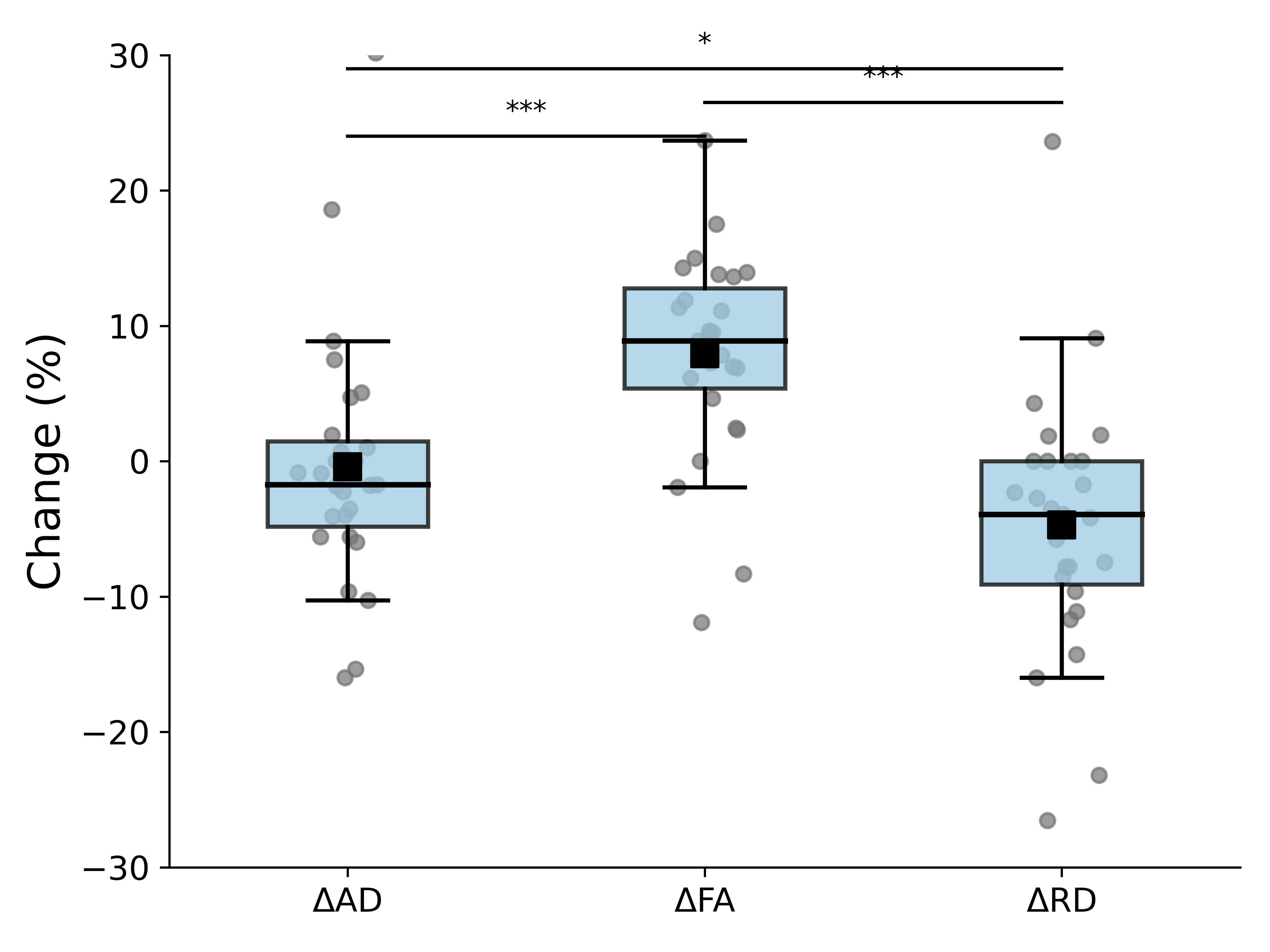

Supplement: Supplementary file 1 [file diagnostics-16-00736-s001.zip › Figure S3.png]
